# HAND2 TRANSCRIPTION FACTOR ENHANCES NMJ ORGANIZATION AND FUNCTION IN OLD MICE

**DOI:** 10.1093/geroni/igz038.327

**Published:** 2019-11-08

**Authors:** Anna Carolina Zaia Rodrigues, Henry J Bonilla, Maria Laura Messi, Zhong-Min Wang, Willard M Freeman, Osvaldo Delbono

**Affiliations:** 1 Wake Forest University, Winston-Salem, North Carolina, United States; 2 University of Oklahoma Health Science Center, Norman, Oklahoma, United States

## Abstract

Over time, declining muscle force and power leads to mobility disability and impaired quality of life. In aging rodents and humans, a denervation and reinnervation process is strongly implicated in sarcopenia: the progressive decline of skeletal muscle mass, composition, and function. We propose that the concomitant decline in expression of Hand2, a key transcription factor (TF) for sympathetic neuron maintenance, induces motor pre- and postsynaptic neuromuscular junction (NMJ) instability and disorganization. To counter the deleterious effect of sympathetic denervation, we developed a novel viral vector (AAV9-Hand2-eGFP, Hand2) carrying Hand2 expression exclusively to sympathetic neurons. Male and female, 16-month-old mice, were examined for signs of muscle denervation and sarcopenia 6 months after IV injection with either Hand2 or control empty virus (AAV9-eGFP, EV). We found that Hand2 increased preterminal synaptic vesicle release, neurofilament phosphorylation (Neurite length: Hand2: 3732±496 µm, EV: 2674±165 µm; P <0.01), NMJ pre/postterminal co-localization, hindlimb muscle mass (EDL: 25%, soleus: 14%, tibialis anterior: 17% and gastrocnemius: 25%; n = 6-8 muscles per treatment group; P < 0.01), myofiber cross-sectional area, and protein kinase-A RIIα/RIα ratio (EV, RIIα:1.05±0.03, RIα:0.93±0.04, ratio: 1.13; Hand2, RIIα:1.81±0.03, RIα:0.94±0.03, ratio: 1.94; P<0.001) which contributes to stability of the NMJ. We also examined Hand2 gene methylation, and RNA-sequencing, muscle metabolomics, and whole body and muscle function with aging in EV and Hand2 injected mice. Our data indicate that expression of Hand2 significantly enhances skeletal muscle adrenergic receptor signaling through the canonical pathway, and prevents in NMJ transmission, and muscle mass and function decline with aging.

